# Dual-Species Fermentation of a *Lycium barbarum*–*Polygonatum cyrtonema* Composite Jiaosu Enhanced Antioxidant Activity and Alleviated Alcohol-Induced Liver Injury in Mice

**DOI:** 10.3390/foods15081435

**Published:** 2026-04-20

**Authors:** Shuyuan Yang, Bingcan Liu, Honghui Geng, Zhen Yu, Wenge Xu, Can Hu, An Zhou, Wencheng Zhang, Zeyu Wu

**Affiliations:** 1 Engineering Research Center of Bio-Process, Ministry of Education, School of Food and Biological Engineering, Hefei University of Technology, Hefei 230601, Chinazwc1012@163.com (W.Z.); 2 School of Materials Science and Engineering, Hunan Institute of Technology, Hengyang 421002, China; 3 The Experimental Research Center, Anhui University of Chinese Medicine, Hefei 230038, China; 4 Intelligent Manufacturing Institute, Hefei University of Technology, Hefei 230051, China

**Keywords:** composite jiaosu, dual-species fermentation, antioxidant activity, oxidative stress, inflammatory cytokines

## Abstract

*Lycium barbarum*–*Polygonatum cyrtonema* composite jiaosu (LBPCJ) was prepared by sequential dual-species fermentation and evaluated in a mouse model of alcohol-induced liver injury. Following process optimization, a yeast-first sequential strategy with intermediate pasteurization was selected, comprising an initial *Saccharomyces cerevisiae* fermentation step, intermediate pasteurization, and a subsequent *Lactiplantibacillus plantarum* fermentation step. Fermentation reduced pH from 4.68 to 3.51 and increased total acidity from 61.06 to 135.39 g LA/L and total phenolic content from 3.01 to 9.39 mg GAE/mL. In vitro antioxidant-related activities were also higher after fermentation, with DPPH, ABTS, and •OH scavenging rates increasing by 39.90%, 29.78%, and 11.10%, respectively. In mice, LBPCJ administration was associated with lower liver index and serum aminotransferase levels, together with attenuated hepatic histopathological alterations, with the high-dose group (15 mL/kg BW) showing the clearest response. These changes were accompanied by higher hepatic SOD and GSH levels and lower MDA, TNF-α, IL-1β, and IL-6 levels. LBJ and PCJ also improved several measured indicators, while LBPCJ showed changes across multiple endpoints under the tested conditions. Overall, sequential fermentation markedly altered the physicochemical and antioxidant-related properties of LBPCJ, and LBPCJ administration improved multiple indicators related to alcohol-induced liver injury in mice, although the specific constituents and underlying mechanisms remain to be clarified.

## 1. Introduction

Alcohol-associated liver disease (ALD) poses a significant global public health concern, primarily arising from long-term and excessive alcohol intake. It encompasses a progressive spectrum of liver injury, ranging from steatosis and alcohol-associated hepatitis to fibrosis and cirrhosis, and may ultimately increase the risk of hepatocellular carcinoma [1]. Recent global statistical data indicate that alcohol consumption was responsible for approximately 2.6 million deaths in 2019, representing 4.7% of total global mortality [2,3]. In China, the burden of ALD has also increased with rising alcohol consumption. A systematic review and meta-analysis covering studies published from 2001 to 2022 reported a pooled prevalence of 4.8%, with an increasing trend over recent decades [4].

Since the liver is the principal tissue responsible for ethanol metabolism, it is highly sensitive to alcohol-induced damage, and alcohol-induced liver injury (ALI) represents an early and experimentally tractable stage in ALD progression. Ethanol metabolism promotes reactive oxygen species (ROS) generation through alcohol dehydrogenase (ADH), aldehyde dehydrogenase (ALDH), and the CYP2E1-dependent microsomal ethanol-oxidizing system (MEOS), thereby contributing to oxidative stress, lipid metabolic disturbance, and mitochondrial dysfunction [5]. In parallel, alcohol disturbs the gut-liver axis by impairing intestinal barrier integrity and altering the gut microbiota, which facilitates the portal translocation of bacterial products such as lipopolysaccharide (LPS) [6]. In the liver, LPS can trigger the activation of the TLR4/NF-κB signaling pathway in Kupffer cells, promoting inflammatory cytokine production, immune cell recruitment, and tissue injury [7,8]. Owing to its multifactorial pathogenesis and marked inter-individual heterogeneity, effective disease-specific therapies for ALD remain limited. Current clinical management still relies primarily on abstinence, nutritional support, and control of complications [9]. Corticosteroids provide only limited short-term benefit in selected patients with severe alcohol-associated hepatitis, while liver transplantation is generally reserved for advanced disease [10].

Given these limitations, dietary interventions and natural products have attracted increasing attention as complementary approaches for the management of ALD [11]. Microbial fermentation is of particular interest because it can alter plant matrices and is often associated with changes in the composition and properties of the final product [12]. Jiaosu, a traditional fermented product derived from fruits, vegetables, and edible-medicinal plants, has therefore attracted growing attention as a plant-derived fermented food matrix. As a complex fermentation system, jiaosu may contain organic acids, polyphenols, polysaccharides, amino acids, and other constituents associated with its overall properties [13]. Fermented plant products have shown favorable results in experimental liver injury models [14,15]. However, direct evidence for jiaosu in ALI-related outcomes remains limited, and head-to-head comparisons between composite jiaosu formulations and their corresponding single-plant products are still scarce.

*Lycium barbarum* and *Polygonatum cyrtonema* are two medicinal and edible plant resources with distinct yet potentially complementary phytochemical profiles. *Lycium barbarum* (*L. barbarum*, commonly referred to as goji berry or wolfberry) is rich in polysaccharides, polyphenols, flavonoids, carotenoids, and amino acids and has been widely reported to exhibit antioxidant, anti-inflammatory, immunomodulatory, and hepatoprotective activities [16,17,18]. Fermented *L. barbarum* products have also shown protective potential in alcohol-related injury models. For instance, lactic acid-fermented goji berry alleviated acute alcohol-induced liver injury and modulated the gut microbiota and its metabolites in mice [19]. *Polygonatum cyrtonema* (*P. cyrtonema*) has likewise been reported to contain polysaccharides, steroidal saponins, flavonoids, and alkaloids [20]. Available evidence indicates that its extracts, particularly the polysaccharide fraction, possess antioxidant, hepatoprotective, anti-inflammatory, and lipid-regulatory activities in experimental models of liver and metabolic disorders [21,22]. Given these compositional differences, combining *L. barbarum* and *P. cyrtonema* may provide a broader substrate basis for fermentation, which could influence the properties of the final product. Previous work has demonstrated that *L. barbarum*–*P. cyrtonema* compound wine can be successfully produced and exhibits favorable in vitro antioxidant properties [23]. However, whether a composite jiaosu derived from these two materials differs from the corresponding single-plant fermented products in vivo ALI-related outcomes remains unclear.

Therefore, this study aimed to develop *Lycium barbarum*–*Polygonatum cyrtonema* composite jiaosu (LBPCJ) through sequential dual-species fermentation and to assess its effects in a mouse model of ALI, with direct comparison to the corresponding single-plant jiaosu preparations derived from *L. barbarum* and *P. cyrtonema*. Intervention outcomes were evaluated using liver injury-related biochemical indices, serum lipid parameters, hepatic oxidative stress-related markers, inflammatory cytokine levels, and histopathological changes, in order to characterize the response patterns of the composite and single-plant fermented preparations across the measured endpoints.

## 2. Materials and Methods

### 2.1. Materials and Reagents

Dried *L. barbarum* fruits with uniform size, intact morphology, and absence of visible defects were sourced from Qinghai Dehao Breeding and Cultivation Co., Ltd. (Qinghai, China). *P. cyrtonema* rhizomes, processed using a traditional nine-cycle steaming-and-drying procedure, were obtained from Jiuhua Mountain, Chizhou City, Anhui Province, China. Starter strains *Saccharomyces cerevisiae* CICC 31735 and *Lactiplantibacillus plantarum* CICC 22696 were acquired from the China Center of Industrial Culture Collection (CICC, Beijing, China). D-glucose was supplied by Hefei Bomei Biotechnology Co., Ltd. (Hefei, China). Rutin and gallic acid standards were supplied by Adamas Life Technology Co., Ltd. (Shanghai, China) and Shanghai Yuanye Biotechnology Co., Ltd. (Shanghai, China), respectively. 2,2-Diphenyl-1-picrylhydrazyl (DPPH) and 2,2′-azino-bis(3-ethylbenzothiazoline-6-sulfonic acid) diammonium salt (ABTS) were bought from Med Chem Express (Monmouth Junction, NJ, USA). HPLC-grade acetonitrile, methanol, ethanol, and n-propanol were obtained from Macklin Biochemical Co., Ltd. (Shanghai, China). All remaining chemicals and reagents were of analytical grade unless otherwise specified.

### 2.2. Preparation of L. barbarum–P. cyrtonema Slurry

*L. barbarum* fruits and *P. cyrtonema* rhizomes were mixed at a mass ratio of 3:2 and homogenized with distilled water at a solid-to-liquid ratio of 1:6 (*w*/*v*) to yield a homogeneous slurry. The initial pH of the slurry was adjusted to 4.6 ± 0.1 using 0.1 mol/L citrate buffer. An enzyme preparation containing cellulase and pectinase (6.03:3.97, *w*/*w*; Shanghai Yuanye Biotechnology Co., Ltd., Shanghai, China) was added at a final concentration of 0.605% (*w*/*v*), and the mixture was hydrolyzed at 40 °C for 1.5 h. Subsequently, the slurry was subjected to ultrasonic-assisted treatment using a SCIENTZ-II D ultrasonic processor (Ningbo Scientz Biotechnology Co., Ltd., Ningbo, China) at 249 W for 10 min. After ultrasonication, the slurry was pasteurized at 65 °C for 30 min, cooled to 25 °C, and used for subsequent fermentation.

### 2.3. LBPCJ Fermentation

*S. cerevisiae* and *L. plantarum* were cultivated in Yeast Extract–Peptone–Dextrose (YPD) broth at 28 °C and de Man, Rogosa and Sharpe (MRS) broth at 37 °C, respectively, until reaching the exponential growth phase. After centrifugation at 3000 rpm and 4 °C for 10 min, cells were collected and rinsed twice using sterile saline solution (0.9% NaCl, *w*/*v*) and resuspended to a final concentration of approximately 8.0 log CFU/mL for inoculation.

For single-strain fermentation, *S. cerevisiae fermentation* (SCF) and *L. plantarum fermentation* (LPF) were optimized separately prior to the establishment of the dual-species fermentation process. For mixed-strain fermentation, four sequential inoculation strategies were designed, namely *S. cerevisiae* followed by *L. plantarum* (SC-LP), *L. plantarum* followed by *S. cerevisiae* (LP-SC), *S. cerevisiae* followed by intermediate pasteurization and then *L. plantarum* (SC-P-LP), and *L. plantarum* followed by intermediate pasteurization and then *S. cerevisiae* (LP-P-SC). In pasteurized sequential fermentation, P denotes an intermediate pasteurization step, during which the broth from the first fermentation stage was heated at 65 °C for 30 min, cooled to 25 °C, and subsequently inoculated with the second microorganism. This step was introduced to terminate microbial activity from the first stage and minimize its interference with the subsequent fermentation stage.

### 2.4. Optimization of Fermentation Conditions

Fermentation conditions were optimized based on strain-specific response indicators during single-strain fermentations. All fermentations were conducted statically in 100 mL flasks, and the working volume was adjusted according to the designated filling ratio. Each experimental condition was performed in triplicate. The filling ratio was defined as the percentage of liquid volume to bottle volume (% *v*/*v*) and was used to regulate the headspace during fermentation. For *S. cerevisiae*, superoxide dismutase (SOD) activity was selected as the response indicator as the objective was to identify fermentation conditions associated with enhanced antioxidant-related functional properties of the product. Accordingly, the effects of inoculation rate (0.5%, 1.0%, 1.5%, 2.0% and 2.5%), fermentation time (12, 24, 36, 48 and 60 h), fermentation temperature (25, 28, 31, 34 and 37 °C), and filling ratio (20%, 30%, 40%, 50% and 60%) on SOD activity were evaluated during process optimization. For *L. plantarum*, viable counts were selected as the response indicator because sufficient bacterial viability is essential for the efficiency and stability of the second-stage fermentation. Therefore, the effects of inoculation rate (1%, 2%, 3%, 4% and 5%), fermentation time (12, 24, 36, 48 and 60 h), and fermentation temperature (28, 31, 34, 37 and 40 °C) on viable counts were assessed in the same manner, with the filling ratio fixed at 30%. After single-strain optimization, four sequential inoculation strategies were compared under their respective optimized conditions using SOD activity as a unified evaluation index. Once SC-P-LP was identified as the preferred fermentation mode, the duration of the *S. cerevisiae* stage was fixed at 24 h, and the duration of the *L. plantarum* stage was further adjusted so that the total fermentation time reached 36, 48, 60, 72, 84 or 96 h. SOD activity and viable cell count were used as evaluation indices.

Viable counts were determined using the plate counting method [24]. Samples were serially diluted in sterile saline, spread onto MRS agar plates, incubated at 37 °C, and results were expressed as CFU/mL. SOD activity was determined using a commercially available assay kit (Nanjing Jiancheng Bioengineering Institute Co., Ltd., Nanjing, China) following the manufacturer’s protocol.

### 2.5. Physicochemical Properties

The pH value was determined using a portable pH meter (PHB-1, Hangzhou Qiwei Instrument Co., Ltd., Hangzhou, China). Total acidity was determined by potentiometric titration using a pH meter according to the National Standard of the People’s Republic of China (GB 12456-2021 [25]), and expressed as lactic acid equivalents (g LA/L). Ethanol concentration was quantified as previously described [26], using an internal standard calibration curve.

### 2.6. Bioactive Components

Reducing sugar (RS) content was determined by the 3,5-dinitrosalicylic acid (DNS) method [27], with a modified sample aliquot volume, and results were expressed as glucose standard equivalents (GE) based on a D-glucose standard curve. Total polysaccharide (TPS) content was assayed by the phenol-sulfuric acid method [28], with modified color development conditions, and results were expressed as glucose equivalents (GE) based on a D-glucose standard curve. Total phenolic content (TPC) was determined using the Folin–Ciocalteu colorimetric method [29], with absorbance measured at 765 nm and results were expressed as gallic acid equivalents (GAE). Total flavonoid content (TFC) was determined using the sodium nitrite-aluminum chloride colorimetric method [30], with a 5 min standing step after NaOH addition, and results were expressed as rutin equivalents (RE).

### 2.7. In Vitro Antioxidant Activities

The in vitro antioxidant capacity of LBPCJ supernatants was investigated using three chemical radical-scavenging assays, namely DPPH, ABTS, and hydroxyl radical (•OH) scavenging. DPPH radical-scavenging activity was determined according to Guo et al. [31], with reaction against 0.1 mmol/L DPPH solution in ethanol at 25 °C in the dark for 30 min and absorbance measured at 517 nm. ABTS radical-scavenging activity was determined with minor adjustments to a previously reported method [32], using an ABTS working solution adjusted to an absorbance of 0.70 ± 0.02 at 734 nm, followed by reaction with diluted supernatant at 25 °C in the dark for 30 min and measurement at 734 nm. Hydroxyl (•OH) radical scavenging activity was evaluated using a modified method [33], based on the salicylic acid-FeSO_4_-H_2_O_2_ reaction system, with incubation at 37 °C for 1 h and absorbance measured at 510 nm. All results were expressed as percentage inhibition relative to the corresponding control and blank.

### 2.8. Animal Experiment

Five-week-old male C57BL/6J mice were purchased from Jiangsu Jicui Yaokang Biotechnology Co., Ltd. (Nanjing, China). All animals were housed under standardized conditions (23 ± 2 °C, 55 ± 5% relative humidity, 12 h light/dark cycle) with ad libitum access to standard chow and water. Following one week of acclimatization, mice were randomly assigned into eight groups (*n* = 6): a control group (CTRL), an alcohol model group (EtOH), a positive control group (Sil), low-, medium-, and high-dose LBPCJ groups (L-LBPCJ, M-LBPCJ, and H-LBPCJ), an *L. barbarum* jiaosu group (LBJ), and a *P. cyrtonema* jiaosu group (PCJ).

The animal experimental protocol was designed with reference to previous studies employing fermented plant-based liquid products in alcohol-induced liver injury models [34,35]. Mice in the CTRL group were orally administered normal saline (10 mL/kg body weight, BW) once daily throughout the experiment. From week 2 to week 5, EtOH and treatment groups received 56% (*v*/*v*) Erguotou liquor (Beijing Erguotou Co., Ltd., Beijing, China) at 10 mL/kg BW by daily gavage. After two weeks of ethanol administration, interventions were initiated and maintained for the final two weeks. Specifically, mice in the Sil group were given silymarin (50 mg/kg BW; Tianjin Xi’en Biochemical Technology Co., Ltd., Tianjin, China) once daily by gavage. Meanwhile, mice in the L-LBPCJ, M-LBPCJ, and H-LBPCJ groups received LBPCJ at 5, 10, and 15 mL/kg BW, respectively, while those in the LBJ and PCJ groups were administered LBJ (10 mL/kg BW) and PCJ (10 mL/kg BW). All test formulations were administered 1 h prior to ethanol gavage, and all gavages were performed at a fixed time each day. Body weight was measured and recorded every 3 days throughout the experimental period.

Following the last gavage, mice were fasted for 12 h while being allowed free access to water, and then anesthetized with isoflurane (Hebei Jindafu Pharmaceutical Co., Ltd., Hebei, China), and blood samples were collected from the orbital venous plexus. Mice were then euthanized, and livers were immediately harvested, rinsed with saline to remove residual blood, dried with filter paper, and weighed. Portions of liver tissue were fixed in 4% neutral paraformaldehyde for histological analysis, and the remaining tissue was snap-frozen in liquid nitrogen and stored at −80 °C for subsequent biochemical analyses. The liver index was calculated as follows:

$$Liver\;index\;(\%)=\frac{liver\;weight}{body\;weight}\times 100$$

All animal experimental procedures were performed in strict compliance with the relevant guidelines for the care and use of laboratory animals, and approved by the Biomedical Ethics Committee of Hefei University of Technology (Approval No. HFUT20251125001).

### 2.9. Biochemical Analysis

Blood samples were stored at 4 °C overnight, followed by centrifugation at 3000 rpm for 15 min at 4 °C to obtain serum. Serum levels of alanine aminotransferase (ALT), aspartate aminotransferase (AST), total cholesterol (TC), and triglycerides (TG) were deterimined using an automated biochemical analyzer (Chemray 800, Shenzhen Rayto Life and Analytical Sciences Co., Ltd., Shenzhen, China) according to the manufacturer instructions. The corresponding commercial assay kits were purchased from the same manufacturer.

Liver tissues were homogenized on ice in 0.9% (*w*/*v*) saline at a tissue-to-liquid ratio of 1:9 (*w*/*v*) to prepare 10% homogenates. After centrifugation at 3000 rpm for 10 min at 4 °C, the resulting supernatants were collected for further biochemical assays. According to the manufacturer protocols, SOD activity, malondialdehyde (MDA) content and reduced glutathione (GSH) level were determined using commercial assay kits (Wuhan Servicebio Technology Co., Ltd., Wuhan, China). Hepatic interleukin-1β (IL-1β), interleukin-6 (IL-6), and tumor necrosis factor-α (TNF-α) levels were quantified using ELISA kits (Thermo Fisher Scientific, Waltham, MA, USA) according to the kit protocols.

### 2.10. Histopathological Analysis

Liver tissues were fixed in 4% neutral paraformaldehyde, processed for paraffin embedding, sectioned, and stained with hematoxylin and eosin (H&E). Histopathological alterations were then observed and evaluated under a light microscope. Semi-quantitative scoring was performed based on previously established liver histology scoring approaches for steatosis and intralobular inflammatory cell infiltration [36,37]. Specifically, each parameter was scored from 0 to 3 (0 = none, 1 = mild, 2 = moderate, 3 = severe), and the total score reflected the overall extent of liver injury.

### 2.11. Statistical Analysis

All determinations were carried out in triplicate, and experimental results are presented as the mean ± standard deviation (SD, *n* = 3). For the animal experiments, data are shown as mean ± SD with six biological replicates (*n* = 6). Statistical processing was performed using GraphPad Prism 10.0 software. Before parametric tests, the Shapiro–Wilk test was applied to verify the normality of data obtained from animal studies. Comparisons among multiple groups were conducted using one-way analysis of variance (ANOVA) accompanied by Tukey’s post hoc test. A *p*-value less than 0.05 was regarded as statistically significant.

## 3. Results and Discussion

### 3.1. Optimization of LBPCJ Fermentation Conditions

**Figure 1:**
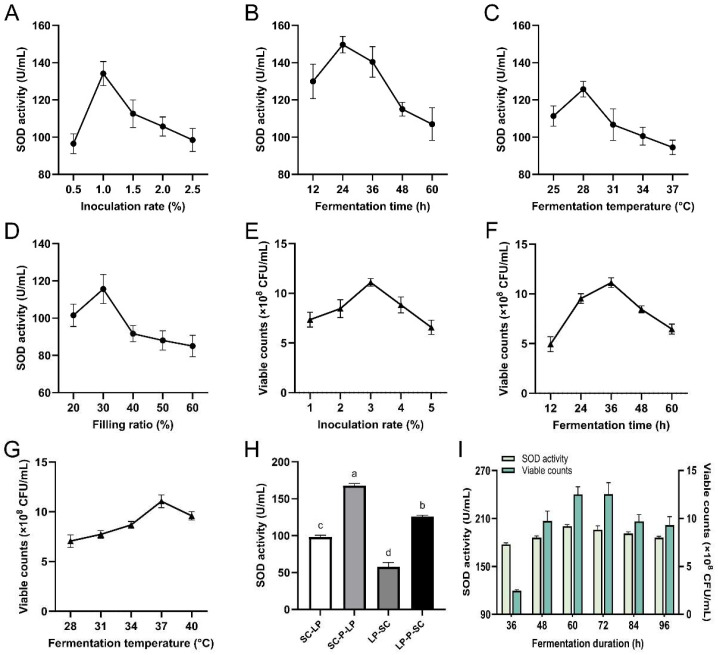
Optimization of fermentation conditions for LBPCJ. Effects of inoculation rate (**A**), fermentation time (**B**), fermentation temperature (**C**) and filling ratio (**D**) on SOD activity during fermentation of *L. barbarum*–*P. cyrtonema* composite slurry by *S. cerevisiae*. Effects of inoculation rate (**E**), fermentation time (**F**), and fermentation temperature (**G**) on viable counts of *L. plantarum*. (**H**) Comparisons of different sequential fermentation strategies based on SOD activity. SC-LP, *S. cerevisiae* followed by *L. plantarum*; SC-P-LP, *S. cerevisiae* followed by pasteurization and *L. plantarum*; LP-SC, *L. plantarum* followed by *S. cerevisiae*; LP-P-SC, *L. plantarum* followed by pasteurization and *S. cerevisiae*. (**I**) Effects of total fermentation duration on SOD activity and viable counts under the SC-P-LP strategy. *S. cerevisiae fermentation* was fixed at 24 h, followed by pasteurization and *L. plantarum fermentation* for varying durations to achieve the designated total fermentation times. Data were expressed as mean ± SD (*n* = 3). One-way analysis of variance (ANOVA) combined with Tukey’s multiple comparisons test was employed to evaluate statistical differences among multiple groups. Different lowercase letters denoted significant differences among groups (*p* < 0.05).

Single-strain fermentation conditions were first optimized using strain-specific response indicators, with SOD activity for the *S. cerevisiae* stage and viable counts for the *L. plantarum* stage (Figure 1A–G). For *S. cerevisiae*, SOD activity increased with inoculation rate and peaked at 1.0% (*v*/*v*), reaching 134.15 ± 6.47 U/mL, and then declined at higher inoculation levels (Figure 1A). SOD activity reached a maximum at 24 h, after which it decreased (Figure 1B), possibly reflecting progressive substrate consumption and metabolite accumulation during fermentation [38]. Temperature significantly influenced the response, with maximal SOD activity of 125.77 ± 4.20 U/mL observed at 28 °C, as shown in Figure 1C. The filling ratio (liquid volume/bottle volume, % *v*/*v*) also exerted a marked effect, with the highest SOD activity at 30% (*v*/*v*) and lower values at higher ratios (Figure 1D), which may be partly related to differences in headspace and oxygen availability under static fermentation conditions [39].

For *L. plantarum*, viable counts increased with the inoculation rate up to 3% (*v*/*v*) and then declined at higher levels (Figure 1E). Similar reductions at excessive inoculum levels have been attributed to accelerated substrate depletion and intensified acidification, which compromise viability in fermented fruit matrices [40]. Viable counts of *L. plantarum* peaked at 36 h, reaching 11.13 ± 0.50 × 10^8^ CFU/mL, and were maximized at 37 °C at 11.05 ± 0.65 × 10^8^ CFU/mL (Figure 1F,G). Comparable temperature ranges have also been reported in plant-based fermentation optimization studies involving lactic acid bacteria [41,42].

Based on the single-strain results, four sequential fermentation strategies were further compared using SOD activity as a unified index (Figure 1H). Among these, the SC-P-LP group exhibited the highest SOD activity (167.91 ± 2.80 U/mL) and was significantly higher than the other tested strategies according to the post hoc comparison, followed by LP-P-SC (125.90 ± 1.67 U/mL) and SC-LP (97.98 ± 2.60 U/mL), whereas LP-SC yielded the lowest value (57.70 ± 5.86 U/mL). These results indicated that both inoculation order and intermediate pasteurization substantially affected the measured fermentation response under the tested conditions. One possible explanation is that different inoculation sequences established different fermentation environments before second-stage inoculation, which may have contributed to the observed differences in SOD activity. Similar differences among yeast-LAB fermentation strategies have also been reported in previous studies [43]. In addition, intermediate pasteurization may have improved stage separation by limiting carryover activity from the first-stage microorganism before inoculation of the second strain. Under the tested conditions, SC-P-LP was therefore selected as the preferred sequential fermentation strategy.

The total fermentation time for SC-P-LP was then determined by monitoring SOD activity and viable counts over time (Figure 1I). SOD activity increased from 36 h, reached a maximum at 60 h, remained at a comparably high level at 72 h, and then declined thereafter. Similarly, viable counts increased sharply from 36 h, peaked at 60–72 h, and decreased at 84–96 h. Accordingly, 60 h was selected as the total fermentation duration for SC-P-LP, as it represented the earliest time point at which SOD activity reached its maximum while viable counts remained within the peak range. This selection balanced the measured response with fermentation time under the tested conditions.

### 3.2. Physicochemical Properties Before and After Fermentation

LBPCJ underwent pronounced physicochemical changes after sequential fermentation (Table 1.). The pH decreased from 4.68 ± 0.04 to 3.51 ± 0.11, total acidity increased from 61.06 ± 2.55 to 135.39 ± 2.16 g LA/L, and ethanol concentration rose from 0.72 ± 0.03% to 2.72 ± 0.13% (*v*/*v*), indicating marked acidification and ethanol accumulation during fermentation. Similar physicochemical changes have been reported in mixed *S. cerevisiae*-*L. plantarum fermentation* systems [43] and in plant-based fermented beverages, in which microbial combination and fermentation strategy affected pH, organic acid composition, ethanol content, and related quality traits [44]. Overall, the marked decrease in pH and increase in total acidity indicate substantial physicochemical changes in LBPCJ during sequential fermentation.

### 3.3. Bioactive Components Before and After Fermentation

**Figure 2:**
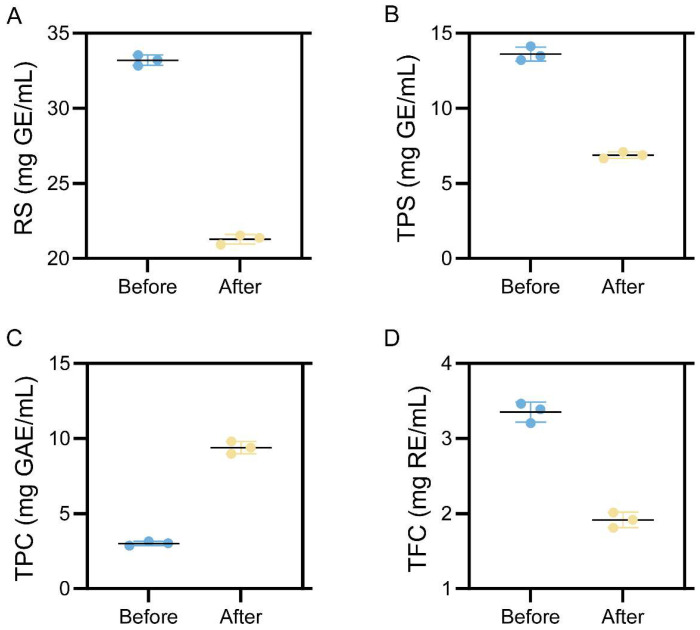
Changes in bioactive components of LBPCJ before and after fermentation. (**A**) reducing sugar (RS), (**B**) total polysaccharide (TPS), (**C**) total phenolic content (TPC), and (**D**) total flavonoid content (TFC). RS and TPS are expressed as D-glucose equivalents (mg GE/mL), TPC as gallic acid equivalents (mg GAE/mL), and TFC as rutin equivalents (mg RE/mL). Data are presented as mean ± SD (*n* = 3).

Carbohydrate-related indices declined after fermentation (Figure 2A,B). Reducing sugar content decreased from 33.20 ± 0.35 to 21.28 ± 0.31 mg GE/mL, consistent with its consumption as a readily available carbon source during microbial fermentation [45]. Total polysaccharide content also decreased from 13.61 ± 0.47 to 6.88 ± 0.21 mg GE/mL, suggesting that fermentation involved not only the utilization of free sugars but also changes in matrix-associated polysaccharides [46]. In contrast, phenolic-related indices changed in opposite directions after fermentation (Figure 2C,D). TPC increased from 3.01 ± 0.15 to 9.39 ± 0.42 mg GAE/mL, whereas TFC decreased from 3.35 ± 0.13 to 1.91 ± 0.10 mg RE/mL. These opposite trends indicate that sequential fermentation was associated with substantial changes in phenolic-related indices, rather than uniform increases across all measured classes.

### 3.4. In Vitro Antioxidant Activities Before and After Fermentation

**Figure 3:**
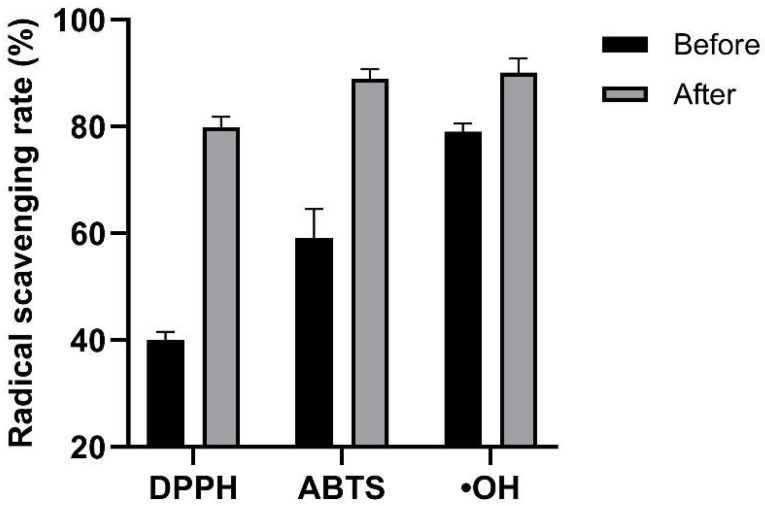
In vitro antioxidant activities of LBPCJ before and after fermentation. DPPH, ABTS, and hydroxyl radical (•OH) scavenging activities were measured to evaluate in vitro antioxidant capacity. Data were presented as mean ± SD (*n* = 3).

Sequential fermentation enhanced the in vitro antioxidant activity of LBPCJ across all radical-scavenging assays (Figure 3). DPPH, ABTS, and •OH scavenging rates increased by 39.90%, 29.78%, and 11.10%, respectively. The greater increases observed in the DPPH and ABTS assays were consistent with the concurrent increase in total phenolic content. In contrast, the smaller increase in •OH scavenging activity may reflect the greater system dependence of hydroxyl radical assays and the fact that relationships between phenolic content and radical-scavenging responses can vary across assay systems and sample matrices [47,48]. Overall, sequential fermentation was associated with improved in vitro antioxidant-related activity of LBPCJ, with the most pronounced enhancement observed in the DPPH and ABTS assay systems.

### 3.5. Effects of LBPCJ on Liver Injury-Related Parameters in Mice with Alcohol-Induced Liver Injury

**Figure 4:**
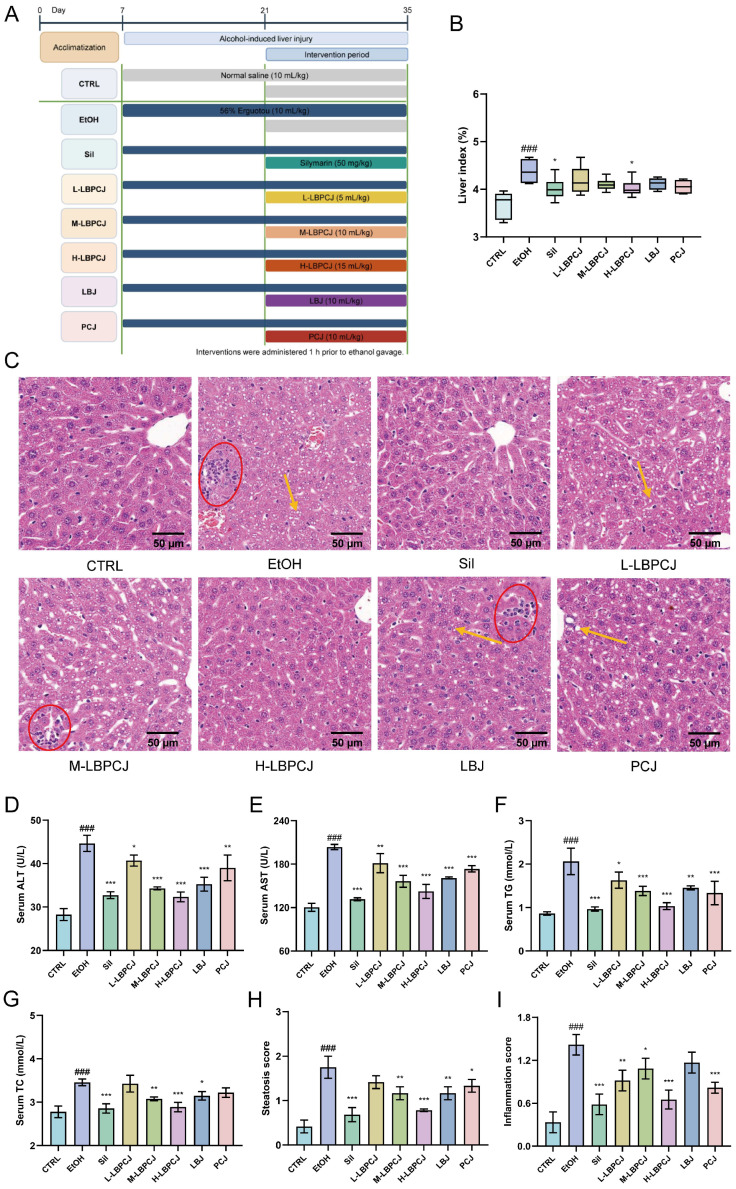
Effects of LBPCJ on hepatic injury in mice with alcohol-induced liver injury. (**A**) Experimental design and treatment timeline. (**B**) Liver index. (**C**) Representative H&E-stained liver sections. Serum ALT (**D**), AST (**E**), TG (**F**), and TC (**G**) levels. (**H**) Steatosis score. (**I**) Inflammation score. Data were presented as mean ± SD (*n* = 6). Statistical differences among multiple groups were analyzed using one-way ANOVA followed by Tukey’s multiple comparisons test. ### *p* < 0.001 vs. CTRL group; * *p* < 0.05, ** *p* < 0.01, *** *p* < 0.001 vs. EtOH group. Red circles indicate intralobular inflammatory cell infiltration, and yellow arrows indicate hepatic steatosis.

Following ethanol challenge (Figure 4A), the EtOH group showed a typical alcohol-induced liver injury phenotype compared with the CTRL group. Liver index was significantly increased (Figure 4B; ###, *p* < 0.001), accompanied by marked elevations in serum ALT and AST (Figure 4D,E; ###, *p* < 0.001), indicating substantial hepatocellular injury. Serum TG and TC were also significantly increased (Figure 4F,G; ###, *p* < 0.001), suggesting disturbed hepatic lipid metabolism after ethanol exposure. Histopathological examination further confirmed these changes: the EtOH group showed obvious hepatocellular steatosis and inflammatory cell infiltration on H&E staining (Figure 4C), together with significant increases in steatosis and inflammation scores (Figure 4H,I; ###, *p* < 0.001). These biochemical and histological alterations confirmed the successful establishment of the alcohol-induced liver injury model.

The silymarin group showed clear improvement, supporting the responsiveness of the model [49]. LBPCJ administration also improved liver injury-related parameters, with the high-dose group showing the clearest overall response. Relative to the EtOH group, H-LBPCJ significantly reduced liver index (*, *p* < 0.05), ALT (***, *p* < 0.001), AST (***, *p* < 0.001), TG (***, *p* < 0.001), and TC (***, *p* < 0.001), as well as steatosis and inflammation scores. M-LBPCJ also produced clear improvements across most endpoints, whereas L-LBPCJ showed weaker effects. Histologically, both medium- and high-dose LBPCJ alleviated hepatocellular vacuolation, steatotic changes, and inflammatory infiltration. LBJ and PCJ also attenuated these abnormalities to some extent. Overall, LBPCJ treatment corresponded to improvement across multiple biochemical and histopathological endpoints in this model.

### 3.6. Effects of LBPCJ on Hepatic Oxidative Stress-Related Indices in Mice with Alcohol-Induced Liver Injury

**Figure 5:**
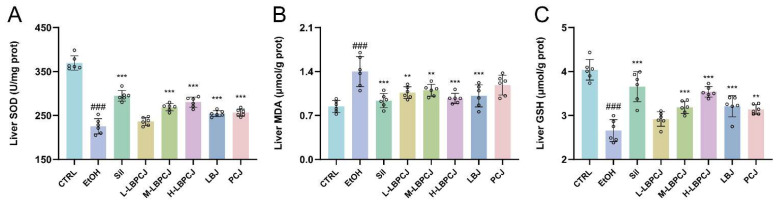
Effects of LBPCJ on hepatic oxidative stress-related indices in mice with alcohol-induced liver injury. Hepatic SOD activity (**A**), MDA content (**B**), and GSH levels (**C**) were determined after the intervention. Data were presented as mean ± SD (*n* = 6). Statistical differences among multiple groups were analyzed using one-way ANOVA followed by Tukey’s multiple comparisons test. ### *p* < 0.001 vs. CTRL group; ** *p* < 0.01, *** *p* < 0.001 vs. EtOH group.

Hepatic SOD activity and GSH content were measured as antioxidant-related indices, whereas MDA was used as an index of lipid peroxidation. Ethanol challenge markedly disrupted hepatic redox homeostasis (Figure 5). Compared with the CTRL group, the EtOH group showed significantly decreased SOD activity and GSH content, together with higher MDA levels (Figure 5A–C; ###, *p* < 0.001), indicating enhanced oxidative stress and lipid peroxidation. Silymarin significantly reversed these changes. LBPCJ supplementation also improved all three indices, with the high-dose group showing the clearest response. Relative to the EtOH group, H-LBPCJ significantly increased SOD and GSH and reduced MDA (***, *p* < 0.001), with changes of approximately +24.3%, +33.2%, and −30.6%, respectively. M-LBPCJ also produced clear improvements, whereas L-LBPCJ was less effective. LBJ and PCJ partially corrected these abnormalities as well. Taken together, LBPCJ treatment coincided with concurrent improvement in all three measured oxidative stress-related indices.

### 3.7. Effects of LBPCJ on Hepatic Pro-Inflammatory Cytokine Levels in Mice with Alcohol-Induced Liver Injury

**Figure 6:**
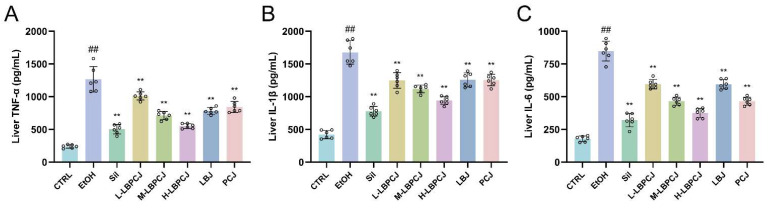
Effects of LBPCJ on hepatic pro-inflammatory cytokine levels in mice with alcohol-induced liver injury. Hepatic levels of TNF-α (**A**), IL-1β (**B**), and IL-6 (**C**) were determined after the intervention. Data were presented as mean ± SD (*n* = 6). Statistical differences among multiple groups were analyzed using one-way ANOVA followed by Tukey’s multiple comparisons test. ## *p* < 0.01 vs. CTRL group; ** *p* < 0.01 vs. EtOH group.

Ethanol challenge significantly elevated hepatic TNF-α, IL-1β, and IL-6 levels compared with the CTRL group (Figure 6A–C; ##, *p* < 0.01), indicating a pronounced inflammatory response in the injured liver. Silymarin significantly reduced all three cytokines. LBPCJ treatment also suppressed hepatic inflammation, with the high-dose group showing the largest reductions. Compared with the EtOH group, H-LBPCJ reduced TNF-α, IL-1β, and IL-6 by approximately 56.5%, 43.9%, and 55.8%, respectively (**, *p* < 0.01). M-LBPCJ also showed clear anti-inflammatory activity, whereas L-LBPCJ was less effective. LBJ and PCJ also lowered these cytokines to some extent. Together with the oxidative stress-related results, these findings indicate that LBPCJ treatment was accompanied by changes in both inflammatory and liver injury-related markers in ethanol-exposed mice.

Taken together, the present results showed that sequential dual-species fermentation markedly altered the physicochemical and compositional characteristics of LBPCJ. The decrease in pH, increase in total acidity, and accumulation of ethanol indicated active microbial transformation of the composite substrate during fermentation, whereas the reductions in reducing sugar and total polysaccharide content were consistent with substantial substrate utilization. Similar shifts in acidity, sugar metabolism, and antioxidant-related properties have also been reported in fruit-based matrices fermented with *Saccharomyces cerevisiae* and *Lactiplantibacillus plantarum*. For example, sequential fermentation of apple juice with *S. cerevisiae* followed by *L. plantarum* altered physicochemical indices and improved antioxidant-related properties [50], whereas different yeast-LAB inoculation strategies in cider produced distinct quality and antioxidant profiles depending on the fermentation mode [43]. In the specific context of *Lycium barbarum*–*Polygonatum cyrtonema* products, Wang et al. reported that fermentation strategy influenced product quality and in vitro antioxidant-related activity in compound wine [23].

The compositional indices and in vitro antioxidant-related assays further showed that fermentation did not affect all measured parameters in the same direction. In the present study, TPC increased markedly, whereas TFC decreased, and these changes were accompanied by higher DPPH, ABTS, and •OH radical-scavenging activities after fermentation. A similar pattern was reported in LAB-fermented jujube-wolfberry composite juice, in which total phenolics increased whereas total flavonoids decreased, together with improved antioxidant capacity [51]. A comparable trend was also observed in jujube juice fermented by *L. plantarum*, where total polyphenols increased while reducing sugar and total flavonoids declined, alongside enhanced biological activity and metabolic profile changes [52]. However, not all fermented fruit systems exhibit the same response pattern. In mulberry wine fermented with *L. plantarum* and *S. cerevisiae*, both phenolics and flavonoids declined during fermentation, despite improvement in some antioxidant-related properties [53]. Recent evidence further indicates that the magnitude and direction of these changes depend strongly on the food matrix, microbial strains, and process conditions [54,55].

In the ethanol-exposed mouse model, LBPCJ treatment improved multiple liver injury-related endpoints. Compared with the EtOH group, LBPCJ, particularly at the high dose, was accompanied by lower liver index, ALT, AST, TG, and TC levels, attenuated steatosis and inflammatory infiltration, higher hepatic SOD and GSH levels, lower MDA levels, and reduced TNF-α, IL-1β, and IL-6 levels. A generally similar response pattern has been reported for fermented goji-based products in alcohol-induced liver injury models [19]. In addition, polysaccharides derived from *P. cyrtonema* have been reported to ameliorate acute alcohol-induced liver damage and to improve oxidative stress- and inflammation-related parameters in mice [22]. In the present study, LBJ and PCJ also improved several endpoints, while LBPCJ showed improvement across multiple measured indicators.

Nevertheless, these findings should be interpreted within the limits of the current study. First, the chemical characterization of LBPCJ remained at the level of global compositional indices rather than compound-specific identification and quantification. Therefore, the present data do not indicate which constituents were altered most substantially during fermentation or which components were most closely associated with the observed in vivo responses. Second, although LBJ and PCJ also improved several endpoints, the present comparative data do not support formal inference regarding superiority or synergism of LBPCJ relative to the corresponding single-plant jiaosu preparations. Third, the parallel changes in fermentation-related composition, in vitro antioxidant-related activity, and in vivo liver injury-related indicators should be interpreted as associative rather than mechanistic. Fourth, the present findings were obtained in an ethanol-exposed mouse model under the current dosing and experimental conditions and therefore should not be directly extrapolated to human alcohol-associated liver disease or longer-term clinical outcomes. Future studies should combine targeted or untargeted chemical profiling with fraction-based validation and pathway-level experiments to identify the fermentation-derived constituents and biological processes most relevant to the observed effects.

## 4. Conclusions

In the present study, LBPCJ was successfully developed through sequential dual-species fermentation. Fermentation markedly altered the physicochemical characteristics of the product and enhanced in vitro antioxidant capacity. In the mouse model of alcohol-induced liver injury, LBPCJ administration improved liver index, serum aminotransferase levels, lipid-related indices, histopathological alterations, hepatic SOD and GSH levels, MDA content, and pro-inflammatory cytokine levels. LBJ and PCJ also improved several of these indicators, whereas LBPCJ showed changes across multiple measured endpoints, with the high-dose group showing the clearest overall response. However, because the present study did not identify specific bioactive compounds or establish mechanistic pathways, these findings should be interpreted as associative rather than causal. The current results provide preliminary experimental evidence supporting further chemical characterization and mechanistic investigation of LBPCJ in the context of alcohol-induced liver injury, but they do not support direct health or functional claims, and extrapolation to human applications requires further validation.

## Figures and Tables

**Table 1:** Physicochemical properties of LBPCJ before and after fermentation.

Parameter	Before Fermentation	After Fermentation
pH	4.68 ± 0.04	3.51 ± 0.11
Total acidity (g LA/L)	61.06 ± 2.55	135.39 ± 2.16
Ethanol concentration (% *v*/*v*)	0.72 ± 0.03	2.72 ± 0.13

Note: Data are expressed as mean ± SD (*n* = 3). Total acidity was expressed as lactic acid equivalents (g LA/L). Results were expressed as ethanol concentration (% *v*/*v*).

## Data Availability

The original contributions presented in this study are included in the article. Further inquiries can be directed to the corresponding authors.
